# *In vitro* Effects of Methylprednisolone Acetate on Equine Deep Digital Flexor Tendon-Derived Cells

**DOI:** 10.3389/fvets.2020.00486

**Published:** 2020-08-05

**Authors:** Stasia N. Sullivan, Nadine N. Altmann, Matthew T. Brokken, Sushmitha S. Durgam

**Affiliations:** Department of Veterinary Clinical Sciences, College of Veterinary Medicine, The Ohio State University, Columbus, OH, United States

**Keywords:** navicular disease, deep digital flexor tendon, methylprednisolone acetate, ECM mRNA expression, collagen, GAG, MMP-3/-13, horse

## Abstract

Primary deep digital flexor tendon (DDFT) pathologies and those accompanying degenerative changes of navicular bone fibrocartilage are major causes of lameness associated with navicular disease. Intrasynovial corticosteroids are mainstay in the treatment due to the anti-inflammatory effects, but their effect on DDFT cell biosynthesis are unknown. The objective of this *in-vitro* study was to investigate the effects of methylprednisolone acetate (MPA) on cells isolated from the dorsal fibrocartilaginous region of forelimb DDFTs (DDFT-derived cells) of 5 horses (aged 11–17 years). Non-adherent aggregate cultures were established from third passage cells over a 72 to 96-h duration prior to treating with medium containing 0 (control), 0.05 and 0.5 mg/mL MPA for 24 h. Tendon and cartilage extracellular matrix (ECM) related gene expression, cell aggregate and culture medium GAG contents, culture medium collagen and MMP-3 and−13 concentrations were measured. After 24 h of treatment, only the higher MPA concentration (0.5 mg/mL) significantly down-regulated tendon ECM related genes; whereas, both MPA doses significantly down-regulated cartilage ECM related genes. MPA treatment did not affect the total GAG content of DDFT-derived cells or total GAG, soluble collagen and MMP-3 and−13 contents in culture medium compared to untreated controls. Future studies to determine the response of DDFT-derived cells with longer exposure times to corticosteroids and in the presence of inflammatory cytokines are necessary. These results are a first step in assessing the effects of intrasynovial medications on equine DDFT, for which currently no information exists.

## Introduction

Navicular disease is a common cause for performance-limiting forelimb lameness in horses. Primary deep digital flexor tendon (DDFT) pathologies and those accompanying degenerative changes of navicular bone fibrocartilage, distal interphalangeal joint and the adjacent peri-ligamentous structures are major causes of lameness associated with navicular syndrome (1–4). Medical treatments consisting of systemic NSAIDs, corrective farriery, and intra-synovial/intra-bursal corticosteroid injections are commonly employed in the clinical management of horses with navicular disease (5–7).

Distal interphalangeal joint and/or navicular bursa injections with corticosteroids, such as triamcinolone acetonide (TA) and methylprednisolone acetate (MPA), are mainstay in treating navicular horses due to their potent anti-inflammatory effects (5–10). In osteoarthritis therapy, intra-articular corticosteroid administration reduces synovitis and articular pain; however, corticosteroids can impair chondrocyte metabolism, resulting in altered extracellular matrix (ECM) composition and subsequent detrimental changes to articular cartilage structure (11–13). Although there is controversy regarding corticosteroid-induced positive (promote glycosaminoglycan (GAG) synthesis, improved cartilage scores) and negative (GAG degradation, cartilage disruption) metabolic effects on articular chondrocytes, recent studies suggest that the disparity in their effects can be attributed to dosage differences than the corticosteroid itself (11–13). Equimolar concentrations of TA and MPA have demonstrated similar effects on chondrocyte ECM transcriptional activity (14–16). While the *in-vitro* and *in-vivo* effects of corticosteroids on articular chondrocyte metabolism have been investigated, their effects on tendon cell biosynthesis are less known. A few studies utilizing human patellar tendon cells have demonstrated that dexamethasone and TA reduced collagen and proteoglycan secretion during *in vitro* culture (17–20). Wei et al. showed that MPA injected into rat sub-acromion space increased collagen type I and type III mRNA expression of infraspinatus tendon tissue (21). To our knowledge, the effects of MPA on equine DDFT-derived cell biosynthetic activities have not been evaluated.

Improvement in advanced diagnostic imaging, primarily magnetic resonance imaging, has demonstrated that DDFT lesions varying from dorsal surface fibrillation, parasagittal splits extending to a variable depth, core lesions, and full-thickness disruption are amongst common pathologies seen in horses with navicular disease (1–3, 7, 22). The DDFT structure at the navicular region is unique in that, it is composed of a superficial dorsal fibrocartilaginous layer, the deep dorsal layer (these 2 regions together constitute the longitudinal layer), and the palmar region comprised of circular collagen bundles. This heterogenous structure represents a functional adaptation of compressive loading from the adjacent navicular bone (3, 23). The cells within in the dorsal fibrocartilaginous zone of the DDFT are “rounded/chondrocyte-like” and secrete a proteoglycan-rich ECM relative to collagen and imparts compressive stiffness to this region (23, 24). Given that these cells synthesize ECM components and maintain tissue integrity, delineating the effects of corticosteroids on their ECM related gene expression and biosynthetic activities is necessary.

The objective of this *in-vitro* study was to investigate the effects of MPA on cells isolated from the dorsal fibrocartilaginous zone of the DDFT at the navicular region (DDFT-derived cells) under non-inflammatory conditions (3). Outcome measures associated with tissue metabolism, such as ECM related gene expression, glycosaminoglycan; GAG and collagen contents, degradation of ECM (MMP-3 and−13) were analyzed. We hypothesized that the concentrations of MPA utilized in this study will have deleterious effects on DDFT-derived cell compared to control DDFT-derived cells.

## Materials and Methods

Forelimb DDFTs were harvested from 5 horses undergoing euthanasia for reasons unrelated to this study. The horses included in this study were 2 mares and 3 geldings (3 Quarter horses, 1 Grade/mixed breed, 1 Hanoverian) between 11 and 17 years of age. All horses had a good body condition score (BCS) >6/9 and were free of forelimb lameness when trotted in-hand on smooth, hard surface. Horses were free of musculoskeletal abnormalities as determined through complete physical examination. The DDFT opposing the navicular bone was determined normal based on gross assessment at the time of harvesting tissue.

### DDFT Harvesting and Cell Isolation

Immediately following euthanasia with sodium pentobarbital (150 mg/kg i.v.), both forelimb digits were disarticulated at the metacarpophalangeal joint. The hoof was cleaned, hair was clipped and the solar surface pared with a hoof knife. The feet were rinsed with water to remove gross debris and the entire digit was scrubbed with disinfectant solution. Each foot was disarticulated at the level of the distal interphalangeal joint to expose the proximal aspect of the navicular bone, without severing the DDFT. Using aseptic technique, the navicular bone was dissected en-bloc from the foot by transecting the surrounding soft tissues and set aside. The DDFT segment directly opposing the navicular bone and 0.5-cm proximal (within the navicular bursa) was harvested using aseptic technique. The dorsal fibrocartilaginous region was dissected from the palmar portion, diced into 0.25 cm^3^ segments and digested in 0.15% collagenase II (Worthington, Lakewood, NJ) in DMEM supplemented with 2% fetal bovine serum (Gemini Biomedicals, Calabasas, CA) at 37°C overnight. Following digestion, the isolated cells were filtered through a 40-um filter (Thermo-Fisher, Waltham, MA). The cells were collected by centrifuging at 300 × g for 5 min. The supernatant was removed, and the cell pellet was resuspended in culture medium containing Dulbecco's modified Eagle's medium containing 4.5 g/l glucose and 300 μg/mL L-glutamine, supplemented with 10% fetal bovine serum, 100 U sodium penicillin/mL and 100 μg streptomycin sulfate/mL (basal expansion medium). Cell yield was determined by use of a hemocytometer, and viability was estimated via trypan blue dye exclusion (Sigma, St. Louis, MO) (25).

### DDFT-Derived Cell Culture

DDFT-derived cells were seeded at 5,000 cells/cm^2^ in monolayer cultures in basal expansion medium and maintained at 37°C, 95% air/5% CO_2_ in a humidified incubator. Non-adherent cells were removed at day 2, the resulting colony forming units were trypsinized at day 7–9 and expanded in monolayers for two passages. Third passage DDFT-derived cells were used in subsequent experiments as described below. DDFT-derived cells were maintained as aggregate cultures to support their chondrogenic phenotype on the basis of results of another study and preliminary data collected in our laboratory (26).

### Aggregate Culture

**Figure 1:**
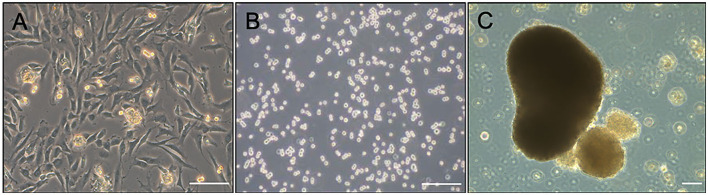
**(A)** Photomicrograph of second passage DDFT-derived cell monolayer culture prior to trypsinization for aggregate culture. **(B)** Dispersed third passage DDFT-derived cells seeded in hydrogel-coated, Ultra Low attachment plates at the start of aggregate culture. **(C)** DDFT-derived cell aggregates formed in 72–96 h of non-adherent culture prior to MPA treatment. Scale bar: 100 μm.

Aggregate cultures were established from third passage DDFT-derived cells isolated from each individual horse (Figure 1A) by resuspending the cells in reduced serum medium (OptiMEM, Thermo-Fisher, Waltham, MA) supplemented with 50 μg/mL ascorbic acid, 100 U sodium penicillin/mL, 100 μg streptomycin sulfate/mL, 1% insulin-transferrin-selenium and 2% fetal bovine serum (27). Cell suspensions containing a total of 3 × 10^6^ cells and 0.5 × 10^6^ cells were aliquoted into each well of a 6- and 24-well hydrogel-coated, Ultra Low Attachment plates, respectively (Corning Inc, Corning, NY) (Figure 1B). Cultures were maintained for 72–96 h while the floating cells formed gross and microscopically visible aggregates (Figure 1C) (27). Fresh medium was supplemented every 36–48 h. Just prior to adding treatments, the medium was collected and frozen at −80°C for later analysis of total soluble collagen and matrix metalloproteinase (MMP) contents. Treatment groups consisted of medium alone (control), medium + 0.05 mg/mL methylprednisolone acetate (0.05 MPA), or medium + 0.5 mg/mL MPA (0.5 MPA). After 24 h of incubation with the aforementioned treatment groups, the media and aggregates were collected separately, snap frozen in liquid nitrogen and stored at −80°C for further analyses. The concentrations of MPA evaluated in this study were based on previous studies measuring corticosteroid-induced biosynthesis in equine chondrocytes (28, 29), corticosteroid dose divided by the average estimated volume of navicular bursa/distal interphalangeal joint (8, 30), and preliminary data collected in our laboratory.

### RNA Isolation and Quantitative RT-PCR

Total RNA was isolated using a previously described protocol (31, 32). Cells from respective aggregate samples in 6-well plates were homogenized in a guanidinium thiocyanate-phenol-chloroform solution reagent (TRIzol, ThermoFisher, Waltham, MA) according to manufacturer's suggested protocol. RNA purity and quantity were assessed spectrophotometrically (Nanodrop, ThermoFisher, Waltham, MA) on the basis of A_260/280_ measurement. Total RNA of 1 μg was reverse-transcribed to cDNA which was used to measure the expression of cardinal cartilage ECM related markers (Sox9, collagen type II, aggrecan) and tendon ECM related genes (collagen type I, collagen type III, COMP) by SYBR green fluorescence-based RT-qPCR; all primer pairs were previously optimized for the mRNAs of interest (Table 1). A total of 10 ηg of cDNA template was utilized for each reaction. Each treatment group from individual horses were run as triplicates and threshold values from the triplicates were averaged prior to calculations. Expression of these genes in DDFT-derived cells treated with control, 0.05 and 0.5 MPA were compared with the expression levels in third passage DDFT-derived cells. Relative gene expression was quantified using the 2^−ΔΔ*C*T^ method, normalized to expression of the reference gene, elongation factor-1α (EF1α) (33).

**Table 1:** Primers for SYBRgreen RT-qPCR.

**Gene**	**Accession Number**		**Sequence**	**Amplicon (bp)**
Col I	NC_009154	S A	5′ GAA AAC ATC CCA GCC AAG AA 5′ GAT TGC CAG TCT CCT CAT CC	231
Col III	AW261123	S A	5′ AGG GGA CCT GGT TAC TGC TT 5′ TCT CTG GGT TGG GAC AGT CT	215
COMP	NM_001081856	S A	5′ TCA TGT GGA AGC AGA TGG AG 5′ TAG GAA CCA GCG GTA GGA TG	223
Sox9	XM_023452130	S A	5′ GAA CGC ACA TCA AGA CGG AG 5′ CTG GTG GTC TGT GTA GTC GT	304
Col II	NM_001081764.1	S A	5′ AGC AGG AAT TTG GTG TGG AC 5′ TCT GCC CAG TTC AGG TCT CT	223
Aggrecan	XM_023650277.1	S A	5′ GAC GCC GAG AGC AGG TGT 5′ AAG AAG TTG TCG GGC TGG TT	202
EF1-alpha	NM_001081781.1	S A	5′ CCC GGA CAC AGA GAC TTC AT 5′ AGC ATG TTG TCA CCA TTC CA	328

### Total Glycosaminoglycan (GAG) Content

The cell aggregates from 0.5 × 10^6^ DDFT-derived cells in control, 0.05 and 0.5 MPA treatment groups were individually digested in papain. Three replicates per treatment group per horse were used for this measurement. Total GAG content of representative aggregates and the culture treatment medium was determined through the use of a 1,9-dimethylmethylene blue binding assay (31, 34). All sample values were compared against a standard curve of chondroitin sulfate values to estimate the total GAG content in paired replicates.

### Total DNA Content

Total DNA content was determined by fluorometric measurement of Hoechst 33258 (Sigma, St. Louis, MO) dye incorporation in DDFT-derived cell aggregates digested with papain that was utilized for GAG assay (31). All measurements (ug) were from 0.5 × 10^6^ DDFT-derived cells in 0.5 mL of the respective culture medium.

### Total Soluble Collagen Content

Total soluble collagen content in each treatment culture medium of DDFT-derived cell aggregates (three replicates per treatment per horse) was measured using a commercially available kit (Biocolor, Carrickfergus, County Antrim, UK) according to manufacturer's recommendations (35). All measurements (ng) were from 0.5 × 10^6^ DDFT-derived cells in 0.5 mL of the respective culture medium.

### Medium MMP-3 and MMP-13 Activity

Matrix metalloproteinase-3 and−13 activity in treatment culture medium (three replicates per treatment per horse) were determined with commercially available ELISA kits (Quantikine, R&D Systems, Minneapolis, MN) in accordance with the manufacturer's protocol. Briefly, the collected control, 0.05 or 0.5 MPA medium samples was combined with MMP-3 or MMP-13 conjugates. The samples were washed and incubated with a substrate solution, and optical density was measured with a microplate reader set at 450 nm.

### Statistical Analysis

Normal distribution of data was assessed by Shapiro-Wilks test. Data were compared with one-way ANOVA (GAG) or the non-parametric equivalent, Kruskal-Wallis test on ranks (chondrocytic gene expression, tendon ECM gene expression, total soluble collagen, MMP-3 and−13). *Post hoc* comparisons for the detection of statistically significant differences between the control, 0.05 and 0.5 MPA treatment groups were conducted with Tukey's method. Differences were considered statistically significant at *P* < 0.05.

## Results

### Tendon ECM Related mRNA Expression

**Figure 2:**
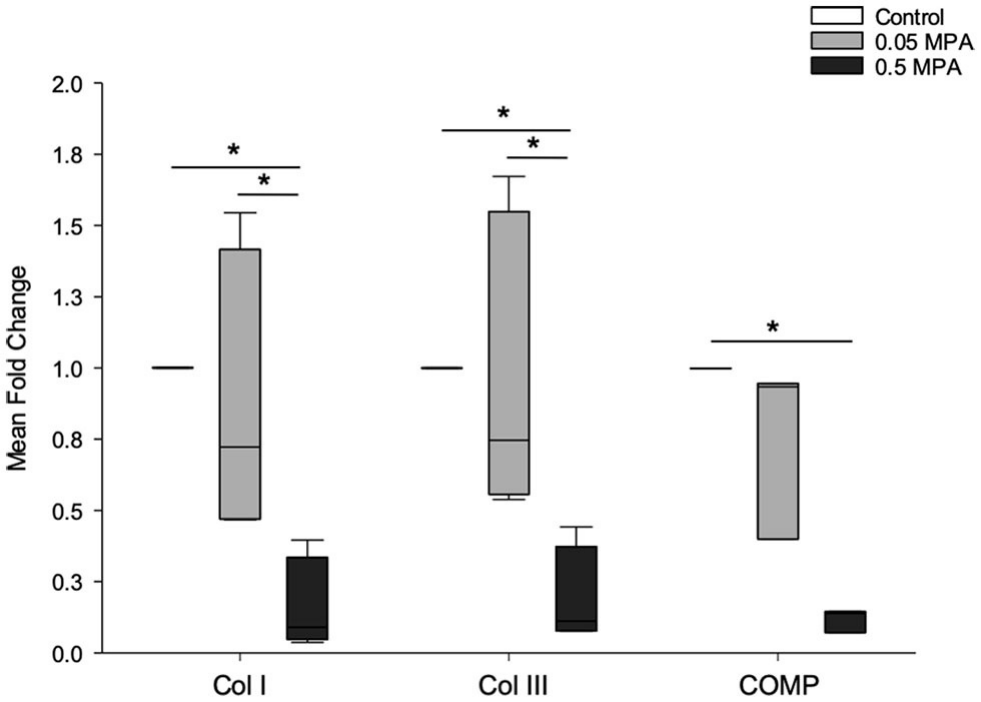
Tendon extracellular matrix (ECM) mRNA expression: Median (and range) fold change collagen type I (Col I), collagen type III (Col III) and cartilage oligomeric matrix protein (COMP) mRNA levels (normalized to EF1a) in DDFT-derived cells after 24 h of treatment with culture media containing 0 mg/mL MPA (Control), 0.05 mg/mL MPA (0.05 MPA) and 0.5 mg/mL MPA (0.5 MPA). *Represents significant difference (*P* < 0.05) between treatment groups.

Mean fold changes in mRNA expression with MPA treatments are depicted in Figure 2. 0.5 MPA treatment significantly decreased collagen type I and collagen type III mRNA compared to 0.05 MPA (5-fold, *P* = 0.013; 4.5-fold, *P* = 0.03) and control (6.25-fold, *P* = 0.013; 5-fold; *P* = 0.036) treatments, respectively. Cartilage oligomeric matrix protein (COMP) mRNA was significantly decreased with 0.5 MPA (8.3-fold, *P* = 0.02) treatment alone. Overall, 0.05 MPA treatment did not significantly affect the tendon related ECM genes of DDFT-derived cells compared to control.

### Cartilage ECM Related mRNA Expression

**Figure 3:**
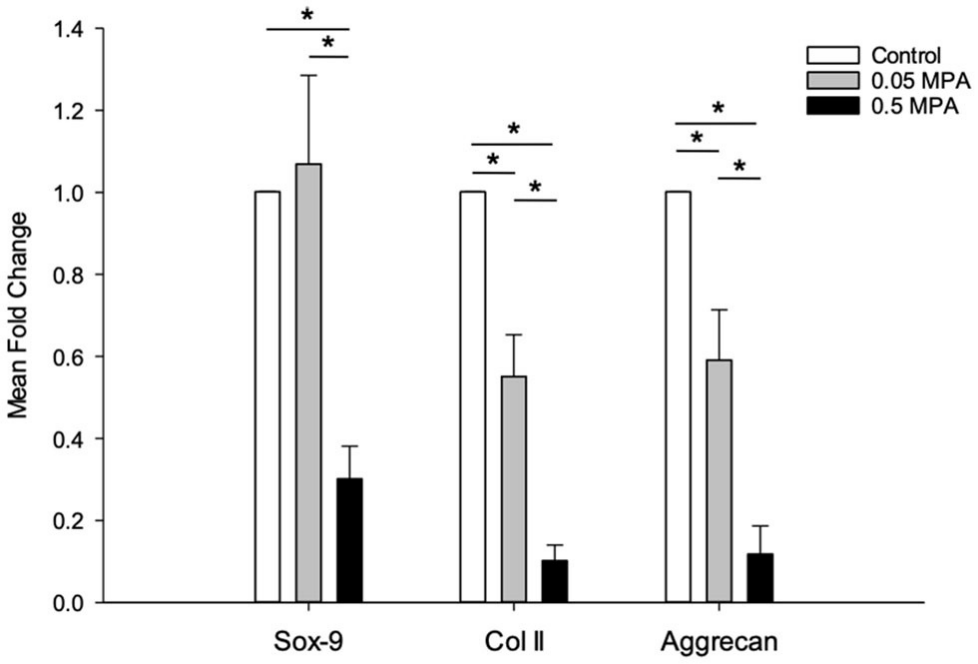
Chondrocytic mRNA expression: Mean (± standard deviation) fold change SRY-box transcription factor-9 (Sox-9), collagen type II (Col II) and aggrecan mRNA levels (normalized to EF1a) in DDFT-derived cells after 24 h of treatment with culture media containing 0 mg/mL MPA (Control), 0.05 mg/mL MPA (0.05 MPA) and 0.5 mg/mL MPA (0.5 MPA). *Represents significant difference (*P* < 0.05) between treatment groups.

0.5 MPA treatment significantly decreased Sox-9, collagen type II and aggrecan mRNA expressions compared to 0.05 MPA (5-, 5.6-, 4.6-fold, *P* = 0.01, 0.003, 0.04) and control (4.5-, 10-, 7.6-fold, *P* = 0.013, 0.005, 0.002) treatments, respectively (Figure 3). In contrast to tendon ECM mRNAs, 0.05 MPA significantly decreased collagen type II and aggrecan mRNAs (2.1-, 1.8-fold, *P* = 0.015, 0.02) compared to control. 0.05 MPA treatment did not significantly (*P* = 0.6) affect Sox-9 mRNA expression of DDFT-derived cells.

### GAG

**Figure 4:**
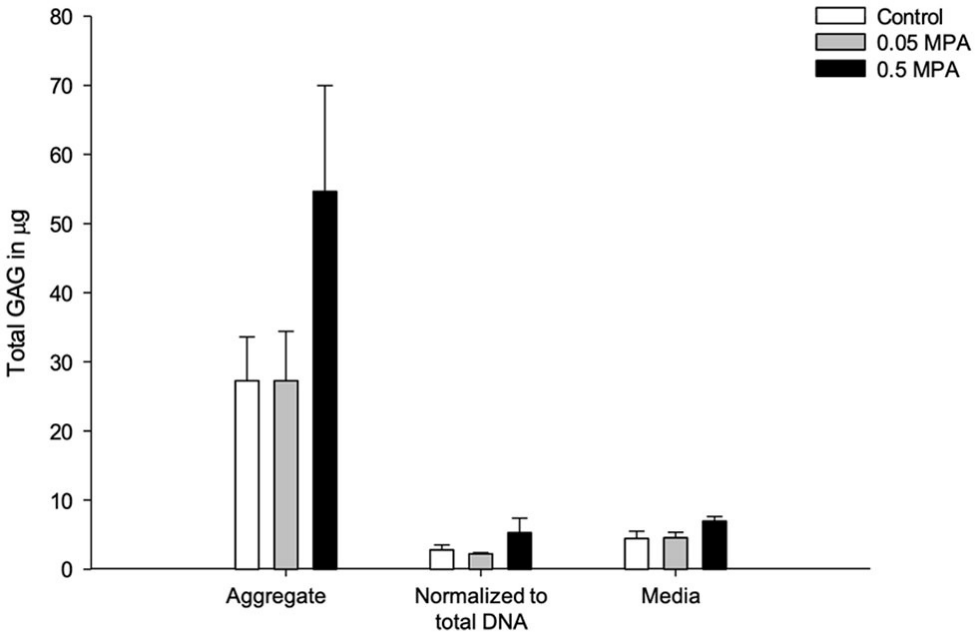
Mean (± standard deviation) glycosaminoglycan (GAG) content (ug) in DDFT-derived cell aggregates, aggregate normalized to total DNA and culture media after 24 h of treatment with 0 mg/mL MPA (Control), 0.05 mg/mL MPA (0.05 MPA) and 0.5 mg/mL MPA (0.5 MPA).

Control and MPA-treated DDFT-derived cell aggregates secreted small amounts of GAG (ug range) during *in-vitro* culture, and was predominantly retained within the aggregates (Figure 4). There was no significant difference (*P* = 0.182) in the total GAG contents of control, 0.05 and 0.5 MPA treated DDFT-derived cell aggregates. This finding remained consistent (*P* = 0.283) when the total GAG content was normalized to cell number (total DNA content). There was no significant difference (*P* = 0.182) in the GAG content released into culture medium of control, 0.05 and 0.5 MPA DDFT-derived cell aggregates.

### Total Soluble Collagen

There was no significant difference (*P* = 0.6) in the total soluble collagen concentrations of culture medium from control, 0.05 and 0.5 MPA treated DDFT-derived cells (Table 2).

**Table 2:** Median (and range) soluble collagen, MMP-3 and MMP-13 concentrations in DDFT-derived cell culture media following treatment with 0 mg/mL MPA (Control), 0.05 mg/mL MPA (0.05 MPA) and 0.5 mg/mL MPA (0.5 MPA).

**Median; range**	**Control**	**0.05 MPA**	**0.5 MPA**	***P*-value**
Soluble collagen (nanograms/mL)	244 (235–343)	240 (236–308)	254 (237–348)	0.9
MMP-3 (nanograms/mL)	0.62 (0.62–0.65)	0.63 (0.62–0.64)	0.63 (0.62–0.65)	0.6
MMP-13 (picograms/mL)	3.32 (3.31–3.32)	3.34 (3.33–3.36)	3.36 (3.35–3.38)	0.6

### MMP-3 and MMP-13

There were no significant differences in the culture medium MMP-3 and MMP-13 concentrations of control, 0.05 and 0.5 MPA treated DDFT-derived cell aggregates (Table 2).

## Discussion

This study's hypothesis that *in vitro* MPA treatment of DDFT-derived cells under non-inflammatory conditions will have negative effects on outcome measures evaluating ECM metabolism was largely not supported by the results of this study. Although MPA treatment of DDFT-derived cells significantly down-regulated tendon and cartilage ECM related genes, ECM metabolism evaluated through culture medium GAG, collagen, MMP-3 and−13 contents was unaffected by MPA treatment. Due to the marked heterogeneity in endogenous cells of DDFT at the navicular region, passage 3 cells isolated from the dorsal fibrocartilaginous region were utilized for all analyses. Given their rounded, chondrocyte-like *in vivo* morphology, DDFT-derived cells were cultured as non-adherent aggregates for 72–96 h prior to MPA treatment. These cells readily aggregated during *in vitro* culture.

Our results demonstrate that after 24 h of treatment, only the higher MPA concentration (0.5 mg/mL) significantly down-regulated tendon ECM relates genes; whereas, both MPA doses significantly down-regulated cartilage ECM related genes. Tendon and cartilage ECM related genes were evaluated given the fibrocartilaginous ECM within the DDFT at this location. Corticosteroid effects on tendon cell transcriptional activities have been minimally investigated. Triamcinolone and dexamethasone decreased collagen and proteoglycan secretion of normal human patellar tenocytes (17–19); however, these studies did not evaluate the changes in ECM transcript levels. *In vivo*, Wei et al. showed that peritendinous MPA injection in normal and injured rat supraspinatus tendon upregulated collagen type I and type III mRNA by 4- to 5-fold (21). Although these studies collectively represent corticosteroid effects on tendon cells, the outcomes reported are not analogous to the equine DDFT given their distinct structural-cellular characteristics. Our findings of down-regulated ECM related genes in MPA-treated DDFT-derived cells is more consistent with normal and interleukin-1β inflamed equine cartilage and articular chondrocytes treated with MPA (16, 36). Further research investigating the differential down-regulation of tendon and cartilage ECM related genes observed in this study is warranted.

MPA treatment did not affect the total soluble collagen content in culture medium, total GAG content of DDFT-derived cells or GAG released into culture medium compared to untreated controls. It should be noted that culture medium collagen and GAG contents measured via standard colorimetric absorbance assays were small, and represents the cumulative sum throughout the aggregate culture duration. It is possible that subtle differences following MPA treatment may have been masked given the assay techniques used here. Other studies that have documented decreased collagen and proteoglycan synthesis in human tenocytes, equine cartilage explants and articular chondrocytes following triamcinolone and MPA treatments utilized ^3^H-proline and ^35^SO_4_ incorporation assays (17–19, 28, 29). Given the low biosynthetic capacities of DDFT-derived cells observed during *in vitro* culture, further dissecting the effect of MPA treatment with radioisotope studies and at longer exposure times are necessary.

Clinical studies have demonstrated transient pain relief and improvement of function following intrasynovial injections in navicular horses (5, 37); however, negative sequelae such as tendon degeneration and rupture have been reported (21, 38). Matrix metalloproteinase (MMP) proteins are involved in tendon ECM breakdown during remodeling in homeostasis and healing and are synthesized by tendon cells, fibroblasts and leukocytes (39, 40). MMP-3 is a potent proteoglycan-degrading enzyme, plays a major role in degrading collagen, fibronectin, elastin and laminin and activates other MMPs such as MMP-1,−7,−9, and−13. Our results show that MPA treatment did not affect the MMP activity of DDFT-derived cells and is likely due to the non-inflammatory culture conditions utilized in this study. MMP-3 expression in normal tendon tissue is low (41). In tendon injury, *in vitro* studies have demonstrated increased (42), decreased (20) or no changes (43) in tissue MMP expression. *In vivo* peritendinous triamcinolone and prednisolone injection in rat Achilles tendon induced MMP-3 expression, tendon cell apoptosis and collagen attenuation (41). Overall our understanding of corticosteroid-induced changes in tendon MMP activity is preliminary and delineating these mechanisms may shed light on structural and functional tendon damage that can occur secondary to corticosteroid administration.

This *in vitro* study has several limitations. The low (*n* = 5) number of horses included combined with low *in vitro* biosynthetic capacity of DDFT-derived cells may have precluded detection of small differences among groups. The lack of an effect by MPA on collagen, proteoglycan contents and MMP production of DDFT-derived cells is likely due to the non-inflammatory culture conditions utilized in this study; similar to the findings of a few studies assessing corticosteroid effects in normal cartilage and chondrocytes (28, 29). It is also not known whether clinically evident DDFT pathology existing in most horses with navicular disease alters sensitivity to corticosteroid-mediated sequalae. Lastly, DDFT-derived cell responses to MPA were only investigated at the 24-h timepoint, and therefore, temporal changes at later timepoints were not evaluated.

Results of this study demonstrate that MPA treatment (concentrations in the range of 10^−5^ M) downregulated ECM related gene expression of equine DDFT-derived cells maintained under non-inflammatory conditions; however, collagen, GAG and MMP contents measured in the culture medium were unchanged. Further studies to assess these responses in the presence of inflammatory cytokines are warranted. While accepting that tissue-level responses under the influence of biomechanical stressors may be different, this is a first step is assessing the effects of intrasynovial medications on equine DDFT for which currently no information exists. These results serve as a foundation for further *in vitro* and *in vivo* studies, and subsequent clinical recommendations.

## Data Availability Statement

The datasets presented in this study can be found in online repositories. The names of the repository/repositories and accession number(s) can be found in the article/supplementary material.

## Ethics Statement

The tendon samples utilized in this study were obtained after euthanizing horses for unrelated reasons and from horses donated to the university for research purposes. As such, an ethical review process specific to this study was not required.

## Author Contributions

SS and SD conducted experiments and analyzed the data. NA contributed to the acquisition, analysis and interpreted the data. SD conceived the study design and obtained funding for the study. All authors wrote, critically revised the manuscript for intellectual content, and approved the final submitted version of the manuscript.

## Conflict of Interest

The authors declare that the research was conducted in the absence of any commercial or financial relationships that could be construed as a potential conflict of interest.
